# Editorial: Nutrition, sarcopenia, and sarcopenic obesity

**DOI:** 10.3389/fnut.2023.1335927

**Published:** 2023-12-13

**Authors:** Nathan A. Berger, Ming Yang, Yoke Mun Chan, Christopher L. Axelrod, Angelos K. Sikalidis, Wen Hu, Lin Kang

**Affiliations:** ^1^Case Comprehensive Cancer Center, Case Western Reserve University, Cleveland, OH, United States; ^2^Center of Gerontology and Geriatrics, West China Hospital, Sichuan University, Chengdu, China; ^3^Department of Dietetics, Universiti Putra Malaysia, Serdang, Selangor, Malaysia; ^4^Integrated Physiology and Molecular Medicine, Pennington Biomedical Research Center, Baton Rouge, LA, United States; ^5^Food Science & Nutrition Department, California Polytechnic State University, San Luis Obispo, CA, United States; ^6^West China Hospital, Sichuan University, Chengdu, China; ^7^Geriatrics Department, Peking Union Medical College Hospital, Beijing, China

**Keywords:** sarcopenia, sarcopenic obesity (SO), muscle mass and function, cancer, diabetes, dietary inflammation index

Sarcopenia, originally identified as loss of muscle mass, has recently been redefined as the independent and/or combined loss of muscle mass, strength, and muscle composition. The latter conceptual approach is associated with lipid deposition in muscle or myosteatosis leading to the pathologic condition of sarcopenia. Interestingly, sarcopenia primarily affects skeletal muscle and may indirectly affect cardiac or smooth muscle.

Sarcopenia is currently considered a public health crisis, estimated to affect 1–30% of community-dwelling individuals, 14–33% of institutionalized patients, nearly 25% of individuals over 65 years of age, and 60% of those over 80. The condition is reported in as many as 50% of patients with cancer, where it may vary with patient age and cancer type. Sarcopenia has been associated with various adverse health outcomes, including frailty, falls, fractures, functional decline, and increased risk of hospitalization and death. In addition to the progressive consequences of sarcopenia, its presence may impair the ability to effectively treat other comorbid conditions.

The development of sarcopenia appears to be multi-factorial and may be caused by multiple processes in addition to aging. These include nutritional deficiency or imbalance, lack of physical activity or sedentary behavior, hormonal imbalance, immunosenescence, mitochondrial dysfunction, inflammatory disorders, chronic diseases, and changes in the intestinal microbiome. Malnutrition (undernutrition, overnutrition, or nutrient deficiencies) contributing to sarcopenia has been proposed for multiple classes of proteins, amino acids, lipids, carbohydrates, and micronutrients including vitamins and minerals.

A particularly unique form of sarcopenia is the muscle loss and weakness that accompanies obesity, termed sarcopenic obesity (SO). This form of sarcopenia is also prevalent worldwide, particularly in older adults and in clinical settings. Individuals with SO are at higher risk of adverse outcomes, such as functional impairment and hospitalization.

The overall goal of this Research Topic of *Frontiers in Nutrition* was to more clearly elucidate the mechanisms contributing to the development of sarcopenia and sarcopenic obesity, to evaluate the impact of sarcopenia on other comorbid conditions, and to identify optimal therapeutic strategies and dietary interventions for the prevention, reversal, and/or management of sarcopenia.

There were 46 manuscripts submitted for this issue, of which 17 were published. The published manuscripts, as described below, focus mainly on the intersection of sarcopenia with other comorbidities including cancer, type 2 diabetes (T2D), cirrhosis, kidney stones, and severe abdominal trauma, among others. Several manuscripts examine the impact of dietary inflammatory indices on the development of sarcopenia, while others explore the importance of nutritional supplementation with and without exercise training.

In a cross-sectional study of 479 individuals with T2D, comprising 264 (55.1%) men and 215 (44.9%) women with a median age of 71 years, Shiroma et al. observed that 8.6% of patients presented with sarcopenia. To evaluate undernutrition status, they compared measurements of serum albumin, GNRI (Geriatric Nutrition Risk Index) based on height, weight, and serum albumin, CONUT (Controlling Nutrition Status) based on serum albumin, total peripheral blood lymphocyte count, and total cholesterol. Their study found the diagnostic power of GNRI to be superior to the measurement of albumin or CONUT for identifying sarcopenia, low skeletal muscle mass index, and low hand grip strength in patients with undernutrition and T2D. The authors discussed the possibility that sarcopenia in T2D may be caused by undernutrition due to therapeutic diets and/or anti-diabetic agents. They suggest the application of the simple GNRI tool to identify patients in need of nutritional support for the improvement of sarcopenia.

Yang Q. et al. reported on a clinical-based observational study of 217 patients (68.6% male), with a mean age of 67.3 ± 11.1 years, exhibiting diabetic foot ulcer (DFU), who were followed over 4 years at Chongqing Medical University. All participants underwent dual-energy X-ray absorptiometry (DXA) to determine body composition. A total of 33 (17.3%) of the 217 patients died. The 5-year survival for the entire group was 58.3%. Patients with sarcopenia had a reduced 5-year overall survival (OS) of 45.9%, compared to 85.4% OS for patients without sarcopenia. Age, sarcopenia, and serum creatinine were independent risk factors for all-cause mortality in these patients with DFUs. The authors suggest that active prevention of sarcopenia may increase survival in patients.

Several groups reported on the impact of sarcopenia in patients with cancer. Two studies focused on sarcopenia in patients with lung cancer and another two in patients with colorectal cancer (CRC). In a study of 126 newly diagnosed stage III and IV lung cancer patients, comprising 97 (77%) men and 29 (23%) women with a mean age of 64.8 ± 8.7 years, Wang et al. reported that the prevalence of sarcopenia and frailty was 25.4% and 32.5%, respectively. They reported the presence of low body mass index (BMI) to be 40% and low skeletal muscle mass measured by bioimpedance analysis (BIA) to be 38.1%. As sarcopenia is associated with chemotherapy toxicity and short survival, they suggest that nutritional intervention should be considered in patients with advanced cancer.

To more specifically evaluate the prognostic significance of sarcopenia in lung cancer patients undergoing targeted or immune therapy, Lyu et al. performed a retrospective analysis of 131 patients with advanced non-small cell lung cancer treated with first-line epidermal growth factor receptor tyrosine kinase inhibitors (EGFR-TKI) or immune checkpoint inhibitors (ICI). Among 35 (26.7%) patients with sarcopenia, median progression-free survival (PFS) and OS were 6.4 and 13 months, respectively, compared to those without sarcopenia, where PFS and OS were 15.1 and 26 months, respectively, and both p < 0.001. Importantly, sarcopenia and non-sarcopenia patients showed similar treatment-related toxicities; however, pretreatment sarcopenia predicted clinical outcomes in patients treated with EGFR-TKIs and ICIs.

Feng et al. evaluated the influence of sarcopenic obesity on treatment complications in patients undergoing radical resections for CRC. Conducting a retrospective cohort study at Beijing Friendship Hospital from January 2017 to May 2018, they evaluated 387 patients, with a median age of 64 years, of whom 63.6% were women, 36.4% were men, 247 had colon cancer, and 140 had rectal cancer. A total of 111 developed surgical complications, and 21 developed medical complications. Sarcopenia and obesity were determined by measuring visceral fat and skeletal muscle mass by CT scan at the L3-L4 intervertebral disc, with sarcopenic obesity defined as a high ratio of visceral fat area/skeletal muscle area. Among these patients, 198 (51.1%) were identified as having SO, while 189 (48.8%) were normal. Patients with SO were of higher age (66 years vs. 62 years), had more T2D, more blood loss at surgery, and showed more vascular invasion of the tumor as compared to their non-SO counterparts. This study showed that sarcopenic obesity is a high-risk factor for post-operative complications, particularly surgical complications. Based on these observations, the authors suggest that strengthening perioperative nutritional status may improve the short-term outcomes of surgery for CRC.

Sarcopenia generally exerts a negative outcome for patients with CRC. However, diagnosis of sarcopenia depends on instrument-based muscle mass measurements such as DXA, computed tomography (CT), or BIA, which are not always clinically available. Gao et al. evaluated the use of serum creatinine/cystatin C ratio (Cr/CysC) as a prognostic indicator in patients with CRC. Creatinine reflects muscle metabolism, whereas cystatin C, a non-ionic protein derived from all nucleated cells, is used to correct for glomerular filtration. This single-center retrospective study from the First Affiliated Hospital, Guangxi Medical University, Nanning, China, analyzed 975 patients (63% men, 37% women, median age 57.5 ± 13.1 years), comprising 494 (51.2%) patients with colon cancer and 476 (48.8%) with rectal cancer. They found 734 patients (75%) with a low Cr/CysC ratio (< 106.25), indicative of low muscle mass, compared to 241 (25%) patients with a high Cr/CysC ratio, indicative of normal muscle mass. A low Cr/CysC ratio was an independent risk factor for PFS and OS in CRC patients. Patients with low Cr/CysC had lower 5-year OS of 52.5% vs. 68.9%. Kaplan–Meier analysis, according to disease stage, showed that patients with stage I-II CRC and low compared to high Cr/CysC ratio had a decreased OS of 67.6% compared to 81.7%. Patients with stage III-IV CRC and low Cr/CysC had a median OS of 36.6% compared to 56.2% in patients with high Cr/CysC ratios. The authors suggest the use of Cr/CysC as a prognostic indicator of sarcopenia in patients with CRC.

Yang H. et al. performed a systematic review examining the impact of sarcopenia in patients with critical illnesses, such as sepsis, trauma, and surgery. The meta-analysis included 38 studies, totaling 6,891 critically ill patients, and observed a 51% pooled prevalence of low skeletal muscle mass (LSMM). Patients with LSMM compared to normal were likely to require mechanical ventilation, at 53.4% vs. 48.9%. Critically ill patients with LSMM compared to normal SMM had a significantly higher mortality risk, with an odds ratio (OR) of 2.35. Based on these studies, the authors indicate the need for early intervention, such as mobilizing patients and providing nutritional support for patients with LSMM.

Sarcopenia was further shown to interact with and impair multiple other conditions. In an analysis of National Health and Nutrition Examination Study (NHANES) data from the periods of 1999–2006 and 2011–2018, Tu et al. showed that among 7,829 participants with hypertension, 47.4% of whom were female and with an average age of 51.4 years, there were 1,352 patients with DXA-determined sarcopenia (17.3%). To assess the relation of dietary inflammatory factors on the development of sarcopenia, the diet inflammatory index (DII) was determined using 24-h dietary recall. Distributing patients from lowest to highest quartiles based on DII showed sarcopenia at 9.4%, 10.9%, 15.3%, and 19.3% of the respective groups. After fully adjusting for potential confounders, those in the highest quartile compared to the lowest quartile showed the highest risk for sarcopenia, with an OR of 2.43, *p* < 0.001. These studies showed that DII is significantly correlated with sarcopenia in patients with hypertension. It is noteworthy that there was no relation found between sex or anti-hypertensive medication as independent risk factors for sarcopenia. The authors stress the importance of the elderly maintaining nutrition with intake of energy, protein, fat, and other substances while maintaining an anti-inflammatory diet to prevent sarcopenia.

Further exploring the relationship between sarcopenia and comorbidities, Zhang et al. used the NHANES database to examine the association between sarcopenia and kidney stones in the adult population of the United States between 2011 and 2018. Among 9,432 participants, they identified 8,793 non-stone formers and 759 stone formers. Among all patients, after propensity matching, there was an OR of 2.365 for the association of kidney stone formers with sarcopenia. In patients of < 40 years of age, the OR was 6.79, whereas for those over 40, the OR was 1.22. The authors conclude that sarcopenia is a potential risk factor for kidney stones in the US adult population.

Seeking to determine the relationship between skeletal muscle mass and nutritional status in patients hospitalized for abdominal trauma in a teaching hospital in China, Xi et al. retrospectively analyzed changes in skeletal muscle mass based on serial L3 CT scans in 103 patients, of whom 91 (88.3%) were male and 12 (11.6%) female, with a mean age of 43.74 ± 15.53 years. They observed a rapid decrease in skeletal muscle mass in week one post-trauma, with subsequent progression indicative of nutritional status and with loss of muscle mass being associated with poor prognosis. The authors indicate the need for further research to optimize recommendations for protein supplementation following acute trauma.

To gain a better understanding of the potential pathophysiologic processes mediating sarcopenia, Liu X. et al. compared plasma metabolomic and unsupervised principal component analyses in 20 patients with hepatitis B virus (HBV)-related cirrhosis and muscle loss to 20 patients with HBV-related cirrhosis without muscle loss and 20 healthy controls. A total of 70 differential metabolites were noted, of which 6 were upregulated and 64 downregulated in patients with muscle loss compared to those without muscle loss. The upregulated metabolites included ethylamine, (r)-3-hydroxybutyric acid, 3—hydroxymethylglutaric acid, 2-ketobutyric acid, 1,5-anhydroglucitol, and creatinine. These metabolites reflected disturbances in 25 pathways, most notably indicating an association of muscle mass loss in patients with cirrhosis, with disordered amino acid metabolism and central carbon metabolism in cancer. Based on these studies, the authors suggest the need to investigate the relationship between citrulline and muscle mass loss in patients with cirrhosis and the potential for the use of citrulline to support the arginine and protein synthesis pathway. Inosine 5′-monophosphate (IMP) was downregulated in patients with muscle loss. Since IMP can serve as a precursor for ATP and GTP, the authors suggest the need to evaluate IMP dietary supplementation to increase energy availability for protein synthesis.

Liu S. et al. provided a comprehensive scholarly review, including discussions of mechanisms and recommendations, for 13 nutritional supplements commonly employed in association with exercise and training to improve the quality of life of patients with sarcopenia. Protein and leucine supplements were recommended to support muscle protein synthesis, as were collagen peptides, although the latter were noted to be no more effective than other protein supplements. Creatine, a natural, non-protein amino acid, was noted to enhance response to exercise training among older adults. β-hydroxy β-methylbutyric acid was not recommended for dietary supplements. The authors suggest that further studies are still needed before recommendations can be made for supplementation with ω-3 fatty acids, inorganic nitrates, probiotics, minerals such as magnesium, selenium, and calcium, and polyphenols.

Ye et al. conducted a systematic review and meta-analysis to address whether dietary supplementation could prevent disuse muscle atrophy in subjects requiring periods of immobility. Examining muscle strength, cross-sectional muscle area, muscle fiber type, distribution, muscle volume, and peak aerobic capacity in 20 randomized control trials (RCTs) comprising 339 subjects, they conclude that dietary supplements with proteins, amino acids, and/or other nutrients, including creatine, ω-3 fatty acids, or β-hydroxy-β-methylbutyrate, extended no protective effect on muscle strength, cross-sectional muscle area, muscle fiber type, distribution, peak aerobic capacity, or muscle volume but did have a protective effect on lean mass. These results further support that nutritional supplementation alone does not prevent loss of muscle mass or strength but rather indicate the need for supplements to be coupled with exercise training to prevent disuse muscle atrophy.

Kim and Park evaluated the effect of ω-3 polyunsaturated fatty acids and dietary fish on the prevalence of low lean mass (LLM) and muscle mass in older women. Analyzing data on 1,620 men and 2,192 women over 65 years of age from the Korean National Health and Nutrition Survey, they found that consumption of EPA, DHA, and fish was negatively associated with the prevalence of LLM but positively associated with muscle mass in older Korean women, although not in men. The authors postulate that the beneficial effects of EPA, DHA, and fish may be associated with their anti-inflammatory effects.

Noting the association of dietary inflammatory potential with inflammatory cytokines, such as interleukin (IL)-1β, IL-4, IL-6, IL-10, tumor necrosis factor- α, and C- reactive protein, Xie et al. conducted a systematic meta-analysis of 24 studies covering 156,536 participants, in which they found that the consumption of diets with high dietary inflammatory index was associated with low skeletal muscle mass and increased risk of sarcopenia, with an OR of 1.53. They suggest the importance of diet strategies with increased intake of anti-inflammatory dietary components (fruits and vegetables) and decreased pro-inflammatory foods (sugar-sweetened beverages and processed meats) to prevent and treat sarcopenia.

To evaluate the potential therapeutic benefits of a complete nutrition drink fortified with anti-inflammatory EPA and branched-chain amino acids (leucine:isoleucine:valine), with the latter employed as muscle synthesis promoters, Khoonin et al. conducted a randomized, blinded placebo, controlled trial of nutrition supplementation and arm muscle exercise in 84 elderly men, with a mean age 65.15–67.86 years and with inadequate protein intake (< 0.5 g/kg/day). After a 3-week intervention, they reported increased IL-10, reduced IL-6, and increased muscle mass and arm strength (measured by hand grip), particularly in patients with complete support; however, differences did not show statistical significance. These promising results suggest the need for randomized, controlled interventions of longer than 3 weeks' duration.

To evaluate a longer intervention, Vijayakumaran et al. conducted a nested, randomized, controlled 12-week pilot study of 16 older Malaysian women with possible sarcopenia measured by low grip strength and/or low 5-times sit-to-stand test, randomized to 3 times per week, undergoing high-intensity program resistance training (PRT) vs. PRT plus multi-nutritional WHEY protein supplementations (PRT + WP). There were six exercises, namely, squats, gluteal kickbacks, seated leg extensions, standing chest press, standing diagonal pull apart, and sectional rows, with exercise progression at 2, 4, and 8 weeks. They observed significant improvement in hand grip strength and stair ascent and overall improvement in patients with possible sarcopenia, but there were no significant differences between groups. The authors reported that the whey protein supplement was readily acceptable with limited side effects. The women indicated increased wellbeing from the intervention and wanted more nutritional information and structured, guided exercise programs. They further indicated their preference for a community-based program.

In conclusion, the research presented in this Research Topic of Frontiers in Nutrition has provided a profound understanding of sarcopenia's intricate relationship with various health conditions. From dietary inflammatory factors to the potential benefits of nutritional supplementation and exercise, this collection of research offers a comprehensive view of the field. These studies underscore the importance of early detection and intervention, emphasizing the role of nutrition in preventing and managing sarcopenia. As we look ahead, future research may explore personalized nutrition strategies, harnessing the potential of precision medicine to tailor optimal interventions for individuals. Additionally, investigating the role of emerging nutritional approaches and exploring innovative exercise regimens will be crucial for optimizing the care of patients with sarcopenia.

## Author contributions

NB: Conceptualization, Project administration, Writing—original draft, Writing—review & editing. MY: Conceptualization, Project administration, Writing—review & editing. YC: Project administration, Writing—review & editing. CA: Project administration, Writing—review & editing. AS: Project administration, Writing—review & editing. WH: Project administration, Writing—review & editing. LK: Project administration, Writing—review & editing.

